# Effects of chronic liver disease on the outcomes of simultaneous resection of colorectal cancer with synchronous liver metastases: a propensity score matching study

**DOI:** 10.3389/fsurg.2023.1184887

**Published:** 2023-09-05

**Authors:** Zheng-Jie Jiang, Xu-Dong Peng, Zheng-Qiang Wei, Gang Tang

**Affiliations:** ^1^ Department of Ultrasound, The First Affiliated Hospital of Chongqing Medical University, Chongqing, China; ^2^ Department of Gastrointestinal Surgery, The First Affiliated Hospital of Chongqing Medical University, Chongqing, China; ^3^ Biliary Surgical Department of West China Hospital, Sichuan University, Chengdu, China

**Keywords:** chronic liver disease, colorectal cancer, liver metastasis, short-term outcomes, surgery

## Abstract

**Introduction:**

Given the rising prevalence of chronic liver disease (CLD), it is increasingly important to understand its impact on surgical outcomes. Our aim was to evaluate the impact of CLD on short-term outcomes in patients with colorectal cancer and synchronous liver metastases undergoing simultaneous surgery.

**Methods:**

We retrospectively reviewed patients with colorectal cancer and liver metastases who underwent simultaneous resection between January 2013 and June 2022. Patients were divided into the CLD and non-CLD groups. Data regarding short-term surgical outcomes were compared between the two groups.

**Results:**

A total of 187 patients were included. After propensity score matching, there were 42 patients in each group, and the basic characteristics of the two groups were similar. Patients with CLD had a significantly greater incidence of postoperative complications (47.6% vs. 26.2%; *P* = 0.042). The operation times of the CLD and non-CLD groups were similar (297 vs. 307.5 min, *P* = 0.537), and the blood loss was comparable between the two groups (250 vs. 155 ml, *P* = 0.066). No significant differences were observed between the two groups in pneumonia (*P* > 0.999), urinary infection rate (*P* > 0.999), ileus rate (*P* = 0.474), wound infection rates (*P* > 0.999), abdominal infection rate (*P* = 0.533), anastomotic leakage rate (*P* > 0.999), digestive hemorrhage rate (*P* > 0.999), bile leakage rate (*P* > 0.999), hepatic hemorrhage rate (*P* > 0.999), reoperation rate (*P* > 0.999), intensive care rate (*P* > 0.999), or severe liver failure (*P* > 0.999). There were no deaths in the two groups. CLD significantly prolonged the length of hospital stay (*P* = 0.011).

**Discussion:**

CLD is an important factor affecting postoperative complications in patients with colorectal cancer liver metastases undergoing simultaneous surgery. Considering the large number of patients with CLD in China, more attention and medical care should be provided to patients with CLD who require simultaneous resection of colorectal cancer with synchronous liver metastases.

## 1. Introduction

Colorectal cancer is one of the most common malignancies and the second leading cause of cancer-related death worldwide (1, 2). Its incidence seems to be associated with smoking, alcohol consumption, obesity, and physical inactivity (3, 4). Due to changes in diet and lifestyle, the incidence of colorectal cancer is increasing (2). Globally, more than 1.8 million new cases of colorectal cancer were reported in 2020. The liver is the most common metastatic target of colorectal cancer, with liver metastasis found in approximately 15% of cases at diagnosis (5). Surgical resection, local ablation therapy, and adjuvant and neoadjuvant therapy are important treatment methods for patients with colorectal liver metastases (6–10). With advances in surgical, adjuvant, and neoadjuvant therapy, more patients with colorectal cancer and liver metastases can undergo radical excision. Laparoscopic surgery can ensure long-term oncological outcomes comparable with open surgery, while providing the advantage of minimally invasive surgery in terms of short-term efficacy (11, 12). Studies have shown that laparoscopic surgery results in similar or lower blood loss, comparable or lower complication rates, and shorter hospital stays compared to traditional open surgery (13). Moreover, mini-incision laparoscopic surgery avoids interruption of the portosystemic shunt and reduces manipulation of the liver when compared to open surgery. In addition, laparoscopic surgery avoids direct exposure of the abdominal cavity to air, thereby reducing the occurrence of electrolyte imbalance (14). The 5-year survival rate for patients with colorectal cancer and liver metastases undergoing radical surgery ranges from 40% to 60% (15).

Chronic liver disease (CLD) is a major health problem worldwide, causing about 2 million deaths yearly (16). In order of importance, the leading causes of CLD in North America and Europe are alcohol-use-related liver disease, non-alcoholic fatty liver disease, and hepatitis C. In contrast, hepatitis B, hepatitis C, alcohol-use-related liver disease, and non-alcoholic fatty liver disease are the main causes of CLD in Asia and Africa (17). Patients with CLD have a higher risk of perioperative complications and death during abdominal, heart, and orthopedic surgery than the risk of those without CLD (18–21). Montomoli et al. (21) found that CLD increased mortality in patients with colorectal cancer within 30 days after surgery. Perioperative complications not only increase the length and cost of hospitalization but also impair the long-term prognosis of patients (22, 23). In addition, postoperative morbidity and mortality were higher in patients who underwent simultaneous primary and metastatic resection compared with patients who underwent colorectal cancer resection alone (5). However, the effect of CLD on perioperative morbidity and mortality in this group of patients is unclear.

We conducted a study to clarify the effect of CLD on the short-term prognosis of radical surgery in patients with colorectal cancer and liver metastasis and to provide evidence for optimizing perioperative management strategies in these patients. Our retrospective cohort study investigated the effect of CLD on postoperative complications and mortality in patients with colorectal cancer and liver metastases undergoing simultaneous resection. We adopted propensity matching score analysis to reduce the influence of potential unbalanced factors.

## 2. Methods

### 2.1. Study population

This single-center retrospective cohort study was conducted at the First Affiliated Hospital of Chongqing Medical University. Patients with colorectal cancer and liver metastases who underwent simultaneous resection between January 2013 and June 2022 were enrolled in this study. The Ethics Committee of the First Affiliated Hospital of Chongqing Medical University approved the study (Approval No. 2022-K534).

The inclusion criteria were 1) age >18 years, 2) colorectal cancer with liver metastasis confirmed by pathology, and 3) history of simultaneous resection for the condition. Patients with a diagnosis of cirrhosis or those who did not undergo radical tumor resection were excluded.

Data regarding patient demographics [age, sex, body mass index (BMI), comorbidity, tumor distance from the anal verge, and neoadjuvant therapy], surgical information (surgical approach, operative time, blood loss, and conversion to open surgery), and postoperative outcomes (length of stay, reoperation, complications within 30 days, readmissions, and mortality) were obtained from the hospital's electronic medical record system. Liver function indices on the day before surgery and 5 days after surgery were collected. We focused on the common causes of CLD, such as chronic hepatitis B, chronic hepatitis C, alcoholic fatty liver disease, and non-alcoholic fatty liver disease. Patients with CLD were included in the CLD group, while the remaining eligible patients were included in the non-CLD group.

### 2.2. Statistical analysis

Statistical analyses were performed using IBM SPSS version 26. The intraoperative conditions, postoperative complications, and postoperative recovery were compared between the two groups. Categorical variables were tested using the *χ*^2^ test or fisher test. The Kolmogorov–Smirnov and Shapiro–Wilk tests were used to determine the data distribution type for continuous variables. Student's *t*-test was used for normally distributed data, and the Mann–Whitney *U* test was used for non-normally distributed data. To reduce the effect of potential confounding factors, sex, age, comorbidity, smoking, neoadjuvant therapy, BMI, and tumor number, size, and stage were matched between the CLD and control groups in a 1:1 ratio using propensity score matching.

## 3. Results

### 3.1. Patients' characteristics

A total of 187 patients with synchronous colorectal liver metastases underwent simultaneous resection from 2013 to 2022. Forty-four (23.5%) patients had CLD, while 143 (76.5%) did not. The majority of patients (*n* = 28, 63.6%) in the CLD group had chronic hepatitis B, while 8 (18.2%), 6 (13.6%), and 2 (4.5%) patients had alcoholic fatty liver, non-alcoholic fatty liver, and chronic hepatitis C, respectively. The baseline characteristics of the enrolled patients are shown in Table 1. The two groups were comparable in terms of sex, age, diabetes mellitus, chronic obstructive pulmonary disease, heart failure, hypertension, smoking, neoadjuvant therapy, BMI, and tumor number, size, and stage. The CLD group had a larger maximum size of liver metastasis than that of the non-CLD group (*P* = 0.015). Patients with CLD had a significantly greater incidence of postoperative complications (47.7% vs. 27.3%; *P* = 0.011).

**Table 1:** Baseline characteristics before propensity score matching.

	Group CLD (*n* = 44)	Group Non-CLD (*n* = 143)	*P*-value
Age (years)^a^	63.5 (51.25–68.5)	61 (53–67)	0.333
Sex (%)			0.708
Male	26 (59.1)	89 (62.2)	
Female	18 (40.9)	54 (37.8)	
BMI (kg/m^2^)^b^	23.44 (3.11)	23.01 (2.68)	0.376
COPD (%)	3 (6.8)	7 (4.9)	0.910
Hypertension (%)	9 (20.5)	23 (16.1)	0.501
Diabetes mellitus (%)	6 (13.6)	17 (11.9)	0.757
Heart failure (%)	4 (9.1)	3 (2.1)	0.092
Smoking (%)	12 (27.3)	34 (23.8)	0.638
ASA			0.951
I–II	25 (56.8)	82 (57.3)	
III	19 (43.2)	61 (42.7)	
T stage (%)			0.504
1–2	4 (9.1)	7 (4.9)	
3–4	40 (90.9)	136 (95.1)	
N stage (%)			0.370
0	16 (36.4)	58 (40.6)	
1	20 (45.5)	49 (34.3)	
2	8 (18.2)	36 (25.2)	
Site of primary colorectal cancer (%)			0.774
Right colon	13 (29.5)	38 (26.6)	
Left colon	14 (31.8)	41 (28.7)	
Rectum	17 (38.6)	64 (44.8)	
Neoadjuvant therapy received (%)	11 (25)	35 (24.5)	0.944
Treatment modality (%)			>0.999
Laparoscopy	44 (100)	141 (98.6)	
Conventional open	0 (0)	2 (1.4)	
Number of liver metastases (%)			0.781
Solitary metastasis	18 (40.9)	67 (46.9)	
2 metastases	13 (29.5)	37 (25.9)	
≥3 metastases	13 (29.5)	39 (27.3)	
Maximum size of liver metastasis (cm)^a^	2.85 (2.00–4.15)	2 (1.3–3)	0.015
Distribution of liver metastases (%)			0.282
Unilobar	25 (56.8)	94 (65.7)	
Bilobar	19 (43.2)	49 (34.3)	

Values in parentheses are percentages, unless indicated otherwise.

BMI, body mass index; COPD, chronic obstructive pulmonary disease; CLD, chronic liver disease.

^a^ Values are median (interquartile range: 25–75th percentile).

^b^ Values are mean (standard deviation).

### 3.2. Surgical results

After matching, 42 patients were included in each group (Table 2). The two groups were comparable in basic characteristics. Surgical outcomes are shown in Table 3. The operation times of the CLD and non-CLD groups were similar (297 vs. 307.5 min, *P* = 0.537), and the blood loss was comparable between the two groups (250 vs. 155 ml, *P* = 0.066).

**Table 2:** Baseline characteristics after propensity score matching.

	Group CLD (*n* = 42)	Group Non-CLD (*n* = 42)	*P*-value
Age (years)^a^	60.3 (12.85)	60.5 (12.03)	0.923
Sex (%)			0.657
Male	26 (61.9)	24 (57.1)	
Female	16 (38.1)	18 (42.9)	
BMI (kg/m^2^)^b^	23.33 (3.06)	23.49 (2.65)	0.783
COPD (%)	3 (7.1)	3 (7.1)	>0.999
Hypertension (%)	7 (16.7)	6 (14.3)	0.763
Diabetes mellitus (%)	6 (14.3)	4 (9.5)	0.500
Heart failure (%)	2 (4.8)	1 (2.4)	>0.999
Smoking (%)	12 (28.6)	9 (21.4)	0.450
ASA			0.503
I–II	24 (57.1)	27 (64.3)	
III	18 (42.9)	15 (35.7)	
T stage (%)			0.356
1–2	4 (9.5)	1 (2.4)	
3–4	38 (90.5)	41 (97.6)	
N stage (%)			
0	15 (35.7)	16 (38.1)	0.769
1	20 (47.6)	17 (40.5)	
2	7 (16.7)	9 (21.4)	
Site of primary colorectal cancer (%)			0.797
Right colon	12 (28.6)	11 (26.2)	
Left colon	14 (33.3)	12 (28.6)	
Rectum	16 (38.1)	19 (45.2)	
Neoadjuvant therapy received (%)	11 (26.2)	12 (28.6)	0.807
Treatment modality (%)			–
Laparoscopy	42 (100)	42 (100)	
Conventional open	0 (0)	0 (0)	
Number of liver metastasis (%)			0.648
Solitary metastasis	17 (40.5)	14 (33.3)	
2 metastases	12 (28.6)	11 (26.2)	
≥3 metastases	13 (31)	17 (40.5)	
Maximum size of liver metastasis (cm)^a^	2.85 (2.00–4.23)	2.95 (1.58–4)	0.737
Distribution of liver metastases (%)			0.826
Unilobar	24 (57.1)	23 (54.8)	
Bilobar	18 (42.9)	19 (45.2)	
Extent of Liver Resection			0.608
Minor resection (<3 segments of liver)	11 (26.2)	9 (21.4)	
Major resection (≥3 segments of liver)	31 (73.8)	33 (78.6)	

Values in parentheses are percentages, unless indicated otherwise.

BMI, body mass index; COPD, chronic obstructive pulmonary disease; CLD, chronic liver disease.

^a^ Values are median (interquartile range: 25–75th percentile).

^b^ Values are mean (standard deviation).

**Table 3:** Operative outcomes and postoperative complications after propensity score matching.

	Group CLD (*n* = 42)	Group Non-CLD (*n* = 42)	*P*-value
Duration of surgery (min)^a^	297 (263.8–361.3)	307.5 (260–386.3)	0.537
Intraoperative blood loss (ml)^a^	250 (122.5–350)	155 (100–262.5)	0.066
Transfusion (%)	2 (4.8)	3 (7.1)	>0.999
Reoperation (%)	0 (0)	1 (2.4)	>0.999
Mortality (%)	0 (0)	0 (0)	–
Intensive care (%)	2 (4.8)	3 (7.1)	>0.999
Conventional open (%)	1 (2.4)	1 (2.4)	>0.999
Hospital stay (days)^a^	12.5 (8–15.3)	8.5 (7–12.5)	0.011
Postoperative complications (%)	20 (47.6)	11 (26.2)	0.042
Bile leakage (%)	1 (2.4)	1 (2.4)	>0.999
Bile duct stenosis (%)	0 (0)	0 (0)	–
Severe liver failure (%)	1 (2.4)	0 (0)	>0.999
Hepatic hemorrhage (%)	1 (2.4)	0 (0)	>0.999
Urinary infection (%)	2 (4.8)	1 (2.4)	>0.999
Pneumonia (%)	2 (4.8)	2 (4.8)	>0.999
Ileus (%)	2 (4.8)	0 (0)	0.474
Wound infection (%)	1 (2.4)	0 (0)	>0.999
Intraabdominal infection (%)	7 (16.7)	5 (11.9)	0.533
Anastomotic leakage (%)	2 (4.8)	2 (4.8)	>0.999
Digestive hemorrhage (%)	1 (2.4)	0 (0)	>0.999

Values in parentheses are percentages, unless indicated otherwise.

^a^ Values are median (interquartile range: 25–75th percentile).

The overall incidence of surgical complications was higher in the CLD group than that in the non-CLD group (47.6% vs. 26.2%, *P* = 0.042). No significant differences were observed between the two groups for pneumonia (*P* > 0.999), urinary infection rate (*P* > 0.999), ileus rate (*P* = 0.474), wound infection rates (*P* > 0.999), abdominal infection rate (*P* = 0.533), anastomotic leakage rate (*P* > 0.999), digestive hemorrhage rate (*P* > 0.999), bile leakage rate (*P* > 0.999), hepatic hemorrhage rate (*P* > 0.999), and severe liver failure (*P* > 0.999). Rates of reoperation (0% vs. 2.4%, *P* > 0.999) and intensive care use (4.8% vs. 7.1% *P* > 0.999) were comparable between the CLD and non-CLD groups, respectively. There were no deaths in either group. The median length of hospital stay was 12.5 days in the CLD group and 8.5 days in the non-CLD group (*P* = 0.011) (Table 3).

Table 4 shows the Clavien–Dindo classification of postoperative complications after propensity score matching. Total bilirubin, direct bilirubin, aspartate transaminase, alanine transaminase, albumin, and prothrombin time before and after surgery were comparable between the CLD and non-CLD groups. The preoperative platelet count (*P* = 0.033) was lower in the CLD group than that in the non-CLD group (Table 5).

**Table 4:** Clavien-Dindo classification of postoperative complication after propensity score matching.

	Group CLD (*n* = 42)	Group Non-CLD (*n* = 42)	*P*-value	*χ* ^2^
Clavien-Dindo classification			0.602	1.860
I	2 (4.8)	0 (0)		
II	12 (28.6)	7 (16.7)		
III	3 (7.1)	1 (2.4)		
IV	3 (7.1)	3 (7.1)		
V	0 (0)	0 (0)		

Values in parentheses are percentages.

**Table 5:** Liver function before and after surgery.

	Group CLD before surgery (*n* = 42)	Group Non-CLD before surgery (*n* = 42)	*P*-value	Group CLD after surgery (*n* = 42)	Group Non-CLD after surgery (*n* = 42)	*P*-value
Total bilirubin (umol/L)^a^	10.4 (7.8–13.5)	9.6 (6.3–11.3)	0.202	11.2 (7.8–17.3)	9.9 (6.9–17)	0.546
Direct bilirubin (umol/L)^a^	3.3 (1.8–5.1)	3.3 (1.5–5.3)	0.809	4.1 (2.6–5.9)	3.4 (2.2–7.5)	0.730
Aspartate transaminase (U/L)^a^	27.5 (21–33.3)	24.5 (17.8–35)	0.393	39.5 (22.8–59)	31 (22–56)	0.471
Alanine transaminase (U/L)^a^	28 (19.8–34.5)	24 (16.3–35.3)	0.543	42.5 (26.5–88.3)	44.5 (22.5–77.5)	0.837
Albumin (g/L)^a^	39.5 (36–45)	39 (36–45)	0.723	33 (30.8–36)	33.5 (29–35)	0.432
Prothrombin time (s)^a^	13.3 (12.9–14.2)	13.2 (12.8–13.8)	0.320	14.3 (13.7–15)	14.2 (13.8–15)	0.946
Platelet (10^9^/L)^b^	207.3 (67.3)	247.5 (99.1)	0.033	184.9 (57.9)	209.1 (85.1)	0.131

^a^ Values are median (interquartile range: 25–75th percentile).

^b^ Values are mean (standard deviation).

## 4. Discussion

Our study found that 23.5% of patients with colorectal cancer and liver metastases had CLD. CLD increased the incidence of postoperative complications, and prolonged hospital stay. However, when comparing patients with CLD to non-CLD patients, we observed a difference in overall postoperative complications, but not in terms of singular or major complications. In addition, CLD had no effect on mortality within 30 days after surgery.

Liver is an important organ for detoxification, protein synthesis, nutrient storage, and immune surveillance (24, 25). Therefore, patients with CLD undergoing surgery have received extensive attention from clinicians. A previous study has reported a 35% incidence of postoperative complications for colorectal cancer (26) and this rate is higher (up to 49%) in patients who undergo simultaneous liver resection and colorectal primary resection (5). Patients undergoing simultaneous resection of primary tumors and liver metastases represent a distinct subset within the population undergoing liver resection. Moreover, the addition of colorectal resection increases the risk of postoperative complications and the cumulative effect of multiple complications is a concern. In this context, not only the extent and type of liver resection, but also the colorectal resection performed can have an important impact. Our study revealed comparable proportions of patients with left colon, right colon, and rectal cancer between the CLD and non-CLD groups. Considering the high postoperative morbidity, our center adopts a multidisciplinary approach to patient selection, indications for surgery, and the simultaneous approach to colorectal liver metastases. Decisions regarding simultaneous surgery are made depends on the size and distribution of liver metastases, the physician's assessment of the safety of surgery, and the patient's preferences and physical condition. If the patient's physical condition is deemed suitable, the residual liver volume is ≥30%–50%, and the surgical procedure on colorectal is not complex, simultaneous surgery is performed. In our cohort, the overall complication rate was 38%. Patients with CLD had a 2.4-fold increased probability of postoperative complications compared with that of patients without CLD. This is similar to the results of some previous studies. A large retrospective study suggested that CLD is a risk factor for postoperative complications in colorectal cancer (20). Ssentongo et al. (27) found that non-alcoholic liver disease was an effective predictor of hyperglycemia and its related complications after major abdominal surgery. In addition, Zaydfudim et al. (28) found that CLD increased complications after hepatectomy. Hamady et al. (29) found that patients with fatty liver had a higher incidence of liver failure after resection for colorectal cancer liver metastases compared with that of patients without fatty liver. This may also be one of the reasons for the shorter postoperative hospital stay in patients without CLD. However, Au et al. (15) explored the effect of a hepatitis B carrier status on surgical outcomes of colorectal cancer liver metastases. Their results showed that the incidence of complications in hepatitis B carrier and non-carrier groups was 9.5% (2/21) and 16.2% (17/105), respectively, but the difference was not statistically significant. However, in the patients enrolled by Au et al. only 22% underwent simultaneous resection of liver metastases. Our study included only patients who underwent simultaneous resection of liver metastases, which may account for the different findings. To the best of our knowledge, previous studies have not treated simultaneous resection of liver metastases and colorectal primary lesions as a separate group, so our results are an important source of evidence.

It is unclear why CLD increases the risk of postoperative complications, and this may be related to the following reasons. First, CLD may affect the action of various drugs, the immune function, and increase the risk of complications such as infection (21). Liver dysfunction can also interact with the anesthetic drugs used during surgery. Mild elevations in serum aminotransferase, alkaline phosphatase, and bilirubin levels are common after surgery under general anesthesia. Although slight elevations in these measures have little effect in patients without CLD, they may lead to an increase in related complications in patients with CLD (19). Second, CLD may affect the recovery of liver function early after surgery, thereby increasing the incidence of complications (30). In addition, CLD is associated with intestinal flora disorder and ectopic intestinal flora, and the latter is one of the important causes of infectious complications after surgery (31, 32).

In addition to being associated with an increased incidence of perioperative complications, CLD may also increase the risk of associated bleeding (20). CLD is associated with decreased coagulation factor levels and thrombocytopenia, which may account for the increased intraoperative blood loss (19, 33). Lu et al. found a 1.6-fold increase in the risk of intraoperative or postoperative blood transfusion in patients with CLD compared with that of patients without CLD during spinal deformity surgery in adults (19). Our study found that the preoperative platelet count was lower in the CLD group than in the non-CLD group. However, there was no significant difference in operation time and intraoperative blood loss between the CLD and non-CLD groups. This may be related to the limited sample size of this study. Therefore, clinicians need to pay more attention to liver function and blood clotting function in this subset of patients. Secondly, chronic liver disease may be associated with thrombocytopenia and increase the risk of bleeding, so preoperative detection and treatment of thrombocytopenia is necessary.

Several studies (20, 28) have shown that CLD increases the risk of death after colorectal cancer surgery and liver surgery. Montomoli et al. (21) reported that patients with liver disease with or without cirrhosis had significantly higher mortality rates within 30 days after colorectal cancer surgery than those of patients without liver disease. In our study, there were no deaths. This may be related to the small sample size of this study. More studies are needed to explore the effect of CLD on mortality after colorectal cancer liver metastasis surgery.

There are some limitations to this study. First, this study was retrospective with some potential bias. Therefore, we used propensity matching score analysis to reduce the influence of unbalanced factors between the two groups. Second, the sample size of this study was limited due to the infrequency of patients with colorectal-cancer-related liver metastases who underwent simultaneous resection. Despite these limitations, this study provides evidence of the impact of CLD on surgery for colorectal-cancer-related liver metastasis, which can help surgeons and healthcare providers develop rational perioperative management strategies in patients with combined CLD.

In conclusion, the present study showed that the presence of CLD was associated with an increased risk of postoperative complications and prolonged hospital stay in patients with colorectal cancer and liver metastases who undergo simultaneous resection.

## Data Availability

The original contributions presented in the study are included in the article/Supplementary Material, further inquiries can be directed to the corresponding author.
